# Mechanisms of Core Chinese Herbs against Colorectal Cancer: A Study Based on Data Mining and Network Pharmacology

**DOI:** 10.1155/2020/8325076

**Published:** 2020-10-27

**Authors:** Tong Lin, Caijun Liang, Wenya Peng, Yuqin Qiu, Lisheng Peng

**Affiliations:** ^1^The Fourth Clinical Medical School, Guangzhou University of Chinese Medicine, Shenzhen 518033, China; ^2^Shenzhen Hospital of Traditional Chinese Medicine, Shenzhen 518033, China

## Abstract

Colorectal cancer (CRC) is now the second most deadly cancer globally. Chinese herbal medicine (CHM) plays an indispensable role in CRC treatment in China. However, the core herbs (the CHs) in the treatment of CRC and their underlying therapeutic mechanisms remain unclear. This study aims to uncovering the CHs and their mechanisms of action of CRC treatment, applying data mining and network pharmacology approach. First, CHM prescriptions treating CRC were collected from clinical studies from the Chinese National Knowledge Infrastructure (CNKI) and MEDLINE databases, and the CHs were identified through data mining. Then, the bioactive compounds and the corresponding putative targets of the CHs were obtained from three traditional Chinese medicine (TCM) databases. CRC related targets were acquired from three disease databases; the overlapping targets between the CHs and CRC were identified as the therapeutic targets. Subsequently, functional enrichment analysis was performed to elucidate the mechanisms of the CHs on CRC. Moreover, networks were constructed to screen the major bioactive compounds and therapeutic targets. Finally, prognostic values of the major target genes were evaluated by survival analysis, and molecular docking simulation was performed to assess the binding affinity of key targets and major bioactive compounds. It came out that 10 the CHs from 113 prescriptions and 190 bioactive compounds with 118 therapeutic targets were identified. The therapeutic targets were mainly enriched in the biological progress of transcription, apoptosis, and response to cytokine. Various cancer-associated signaling pathways, including microRNAs, TNF, apoptosis, PI3K-Akt, and p53, were involved. Furthermore, 15 major bioactive compounds and five key target genes (VEGFA, CASP3, MYC, CYP1Y1, and NFKB1) with prognostic significance were identified. Additionally, most major bioactive compounds might bind firmly to the key target proteins. This study provided an overview of the anti-CRC mechanisms of the CHs, which might refer to the regulation of apoptosis, transcription, and inflammation.

## 1. Introduction

Colorectal cancer (CRC), including colon and rectal cancer, is the third most frequently diagnosed cancer and the second leading cause of cancer-related deaths worldwide [1]. Furthermore, the rising incidence of CRC at younger ages (<50 years-old) is a merging trend [2]. Hereditary accounts for approximately 10–20% of all CRC patients, while 60–65% of CRC cases arise from modifiable risk factors, such as smoking, red and processed meat intake dietary, excessive alcohol intake, and obesity [3]. Currently, surgical resection is still the only curative treatment for CRC patients. Nevertheless, due to its occult onset, most CRC patients are diagnosed in advanced stages when surgery is unavailable [4]. Another issue is that major complications occur on up to 15% patients, and postoperative recurrence and metastasis are quite common [4, 5]. Neoadjuvant and adjuvant chemoradiotherapy is now generally accepted standard treatment for locally advanced CRC, with the aim of reducing recurrence and metastasis [6–8]. Disappointing, long-course chemoradiotherapy brings adverse reactions, such as myelosuppression, cumulative neuropathy, gastrointestinal tract reaction, and organs damage, which badly reduce the quality of life of the patients, and even leads to interruption of therapies [9]. Additionally, chemoradiotherapy is often confronted with treatment resistance [10].

Chinese herbal medicine (CHM) plays an indispensable role in integrative therapy of malignancies in China. It has been widely reported that CHM prescriptions could improve survival and quality of life of CRC patients through the following effects: (1) preventing tumorigenesis, suppressing tumor growth, and reducing metastasis and recurrence [11–14]; (2)increasing sensitivity and alleviating side effects of chemo- or radiotherapy; (3) relieving tumor-related symptoms or surgery complications, such as fatigue, pain, loss of appetite, diarrhea, nausea, and vomiting; and (4) improving immunity, lessening the damages induced by conventional treatments, and ameliorating bone marrow suppression [9, 15–18]. However, CHM prescriptions in the treatment of CRC differ a lot, due to varied educational backgrounds and personal clinical experiences of different traditional Chinese medicine (TCM) doctors. The underlying patterns or the core herbs (the CHs) of the prescriptions and the bioactive compounds and therapeutic mechanisms of the CHs are still in veil.

The application of data mining and machine learning can help people better understand the patterns of herbs use from abundant clinical prescriptions [19]. CHM effects in a multicomponent, multitarget, and multipathway mode, which makes it unique superiority in treating complex diseases, but causes difficulty in clarifying mechanisms of action; meanwhile. Integrated systems biology, bioinformatics, and poly-pharmacology, network pharmacology provides an effective solution to uncover the synergistic effects and underlying mechanisms of multicomponent and multitarget agents through network analysis [20, 21]. Molecular docking is an in silico structure-based method to predict ligand-target interactions at a molecular level, which has been widely used in drug discovery [22].

**Figure 1:**
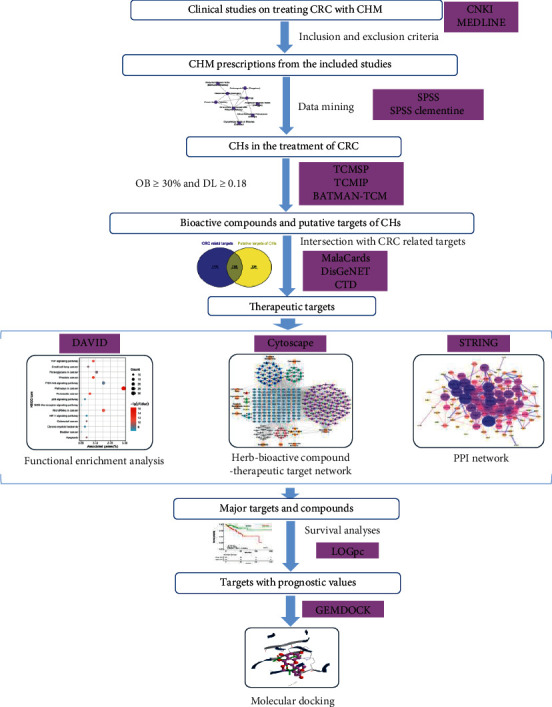
A flowchart of this study.

In the present study, the CHs in the treatment of CRC were identified through data mining from prescriptions in clinical studies. Then, the bioactive compounds, the putative targets, and mechanisms of the CHs acting on CRC were investigated by network pharmacology approach. Finally, survival analysis and molecular docking simulation were performed to strengthen the results of network pharmacology analysis. A flowchart of this study is described in Figure 1.

## 2. Materials and Methods

### 2.1. Inclusion and Data Mining of CHM Prescriptions

Clinical CHM prescriptions in the treatment of CRC were collected from studies from the Chinese National Knowledge Infrastructure (CNKI) and MEDLINE databases. The literature search was conducted in topic, title, and abstract, using the following search terms: (“traditional Chinese medicine”) OR (“Chinese herb”^*∗*^) OR (decoction) OR (prescription) AND (colorectal cancer) AND (clinical); the timespan was set from database creation to 1st June 2020.

The inclusion criteria for studies were as follows: (1) the first diagnosis of patients which was CRC, (2) clinical studies about oral CHM prescriptions treating CRC, combined with (chemo)radiotherapy or not, and (3) the prescriptions should be effective. Effectiveness is defined as statistically significant protective effects in the CHM treatment group, compared with the control group. The protective effects included but are not limited to the alleviation of cancer-related symptoms and/or adverse reactions of (chemo)radiotherapy, prolongation of survival, improvement of quality of life, and immunity. The exclusion criteria were as follows: (1) review articles and laboratory experimental studies, (2) the constituent herbs in prescriptions not published in full, (3) studies in which the patients were treated with extract (s) of a single herb or Chinese patent medicine, and (4) the number of patients in any single group being less than 20.

The CHM prescriptions were extracted from the eligible studies, and a database was formed. Then, frequency analysis was performed with SPSS software (version 21.0, IBM Corp., Armonk, NY, USA). And association rule analysis was conducted by a priori algorithm with SPSS Clementine software (version 12.0, SPSS Inc., Chicago, IL, USA), to identify the CHs from all prescriptions. In this study, association rules were set under the condition of support degree ≥20% and confidence degree ≥50%.

### 2.2. Bioactive Compounds and Putative Target of the CHs

The compounds of all the CHs were collected from Traditional Chinese Medicine Systems Pharmacology Database and Analysis Platform (TCMSP, http://tcmspw.com/tcmsp.php) [23], Integrative Pharmacology-based Research Platform of TCM (TCMIP, http://www.tcmip.cn/) [24], and Bioinformatics Analysis Tool for Molecular Mechanisms of Traditional Chinese Medicine Database (BATMAN-TCM, http://bionet.ncpsb.org/batman-tcm/) [25].

Based on the TCMSP database, oral bioavailability (OB) ≥ 30% and drug-likeness (DL) ≥ 0.18 were set as the screening criteria of bioactive compounds, which were labeled as “Mol ID” in the TCMSP database in the further study [26]. The putative targets corresponding to the bioactive compounds were collected from the above three TCM databases, and all target names were converted into gene symbols using the UniProt database (https://www.uniprot.org/) for the subsequent study.

### 2.3. Therapeutic Targets of the CHs in the Treatment of CRC

CRC related targets were obtained from the MalaCards (https://www.malacards.org/) [27], DisGeNET (http://www.disgenet.org/) [28] and the Comparative Toxicogenomics Database (CTD, http://ctdbase.org) [29], and the search terms used were “colorectal carcinoma” or “colorectal cancer.” The overlapping targets between the CHs and CRC were determined using Venn diagram with an online tool named Venny 2.1 (https://bioinfogp.cnb.csic.es/tools/venny/index.html). And the overlapping targets were recognized as the potential therapeutic targets of the CHs in CRC.

### 2.4. Functional Enrichment Analysis

To elucidate the therapeutic mechanisms of the CHs on CRC, gene ontology (GO) functional enrichment analysis and Kyoto Encyclopedia of Genes and Genomes (KEGG) pathway enrichment analysis of therapeutic targets were performed using the Database for Annotation, Visualization, and Integrated Discovery database (David, https://david.ncifcrf.gov/) [30]. The results with *P* value <0.05 and false discovery rate (FDR) < 0.05 were considered significant, which were visualized using the GraphPad Prism software (version 6.0, San Diego, CA, USA) and “ggplot2” package in R.

### 2.5. Network Construction, Major Compounds, and Major Targets Screening

Two networks were constructed in this study. (1) A herb-bioactive compound-therapeutic target network was established to show the interactions among the CHs, bioactive compounds, and therapeutic targets. (2) A protein-protein interaction (PPI) network was built to explore the interactions among therapeutic targets. The PPI data of therapeutic targets were analyzed using Search Tool for the Retrieval of Interacting Genes/Proteins platform (STRING, https://string-db.org/) [31]. The species was set as “*Homo sapiens*”, and the minimum confidence score was set as 0.9.

The diagrammatic networks were constructed using Cytoscape software version 3.7.2 [32], and the major bioactive compounds and targets were screened by network topological parameters calculated with Network Analyzer plug-in. The topological parameter of degree value was used to describe the importance of a node in a network, which is defined as the number of edges linked to a certain node. A major node must have a degree value two times greater than the median degree value of all nodes in the network [33].

### 2.6. Survival Analysis of the Major Target Genes

The prognostic value of major target genes was assessed by survival analysis using a web server named Long-Term Outcome and Gene Expression Profiling Database of Pan-Cancers (LOGpc, http://bioinfo.henu.edu.cn/DatabaseList.jsp), which provides 13 types of survival terms for 28,098 patients from 26 malignancies [34]. All cases were categorized into high and low expression groups by the median expression of a certain therapeutic target gene; then overall survival (OS) and disease free survival (DFS) were analyzed by log-rank test. Kaplan–Meier (KM) curves, hazard ratio (HR), 95% confidence intervals (CI), and log-rank *P* value were generated online. The results with *P* < 0.05 were considered statistically significant, and the targets correlated with patients' survivals were considered as the key targets.

### 2.7. Molecular Docking Simulation

To evaluate the binding potential of the major bioactive compounds of the CHs with the key target proteins, molecular docking simulation was performed using GEMDOCK software (version 2.1, National Chiao Tung University, Hsinchu, Taiwan) [35]. The empirical scoring function of GEMDOCK is as follows: Fitness = van der Waal energy + hydrogen bonding energy + electro statistic energy. A fitness value was used to estimate the binding affinity of a protein and a ligand and the fitness value of the corresponding protein-original ligand complex was used as a comparison. The generic evolutionary method parameters were set as population size = 200, generations = 70, and number of solutions = 2. All 3D crystal structures of target protein-original ligand complexes were downloaded as “.pdb” files from Protein Data Bank (PDB, http://www.rcsb.org/pdb/) [36]. All 3D molecular structures of original ligands and major bioactive compounds were downloaded as “.mol2” files from TCMSP database or ZINC database (http://zinc.docking.org) [37].

## 3. Results

### 3.1. Results of Data Mining

**Figure 2:**
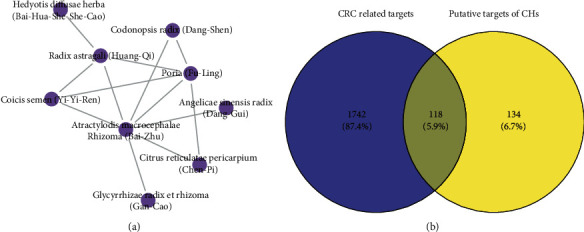
The association rules network of the CHs and the Venn diagram; (a) the association rules network of the CHs. An edge represents an association rule between two herbs, which met the criteria of support degree ≥20% and confidence degree ≥50%, and (b) the Venn diagram showing overlapped targets between the CHs and CRC.

1130 and 131 studies were obtained from CNKI and MEDLINE databases, respectively. According to the inclusion and exclusion criteria above, 108 and 3 clinical studies were included from CNKI (published in Chinese) and MEDLINE (published in English) databases, respectively. A list of the clinical studies included in this study is provided in Supplementary Table 1. Then, a total of 113 CHM prescriptions treating CRC were extracted from the 111 clinical studies. There were 196 different Chinese herbs used in all prescriptions, and the frequency analysis of herbs revealed that the ten most frequently used Chinese herbs were Atractylodis Macrocephalae Rhizoma (Bai-Zhu), Poria (Fu-Ling), Radix Astragali (Huang-Qi), Glycyrrhizae Radix et Rhizoma (Gan-Cao), Codonopsis Radix (Dang-Shen), Coicis Semen (Yi-Yi-Ren), Citrus Reticulatae Pericarpium (Chen-Pi), Hedyotis Diffusae Herba (Bai-Hua-She-She-Cao), Angelicae Sinensis Radix (Dang-Gui), and Scutellariae Barbatae Herba (Ban-Zhi-Lian), which were recognized as the CHs in the treatment of CRC. The usage frequency of each CH is shown in the second and third columns of Table 1. Besides, the association rule analysis revealed that nine herbs out of the CHs were often used together, whose associations are shown in Figure 2(a).

### 3.2. Bioactive Compounds and Putative Therapeutic Targets of the CHs

The eligible bioactive compounds of every herb were merged from three TCM databases, bioactive compounds reiterated, or without putative targets were removed. Totally, 190 bioactive compounds and 252 putative targets of the CHs were included. The final counts of bioactive compounds of each CH are shown in the fourth column of Table 1, and duplicates existed among different the CHs.

In terms of CRC related targets, 838 targets were obtained from MalaCards, and the top 1000 targets were collected from DisGeNET and CTD databases, according to their rank of inference score, respectively. A total of 1860 CRC related targets were found after removing the duplicates. Subsequently, 118 overlapping targets between the CHs and CRC were identified using the Venn diagram (Figure 2(b)), which were considered as the potential therapeutic targets of the CHs for CRC.

### 3.3. GO and KEGG Pathway Enrichment Analysis

**Figure 3:**
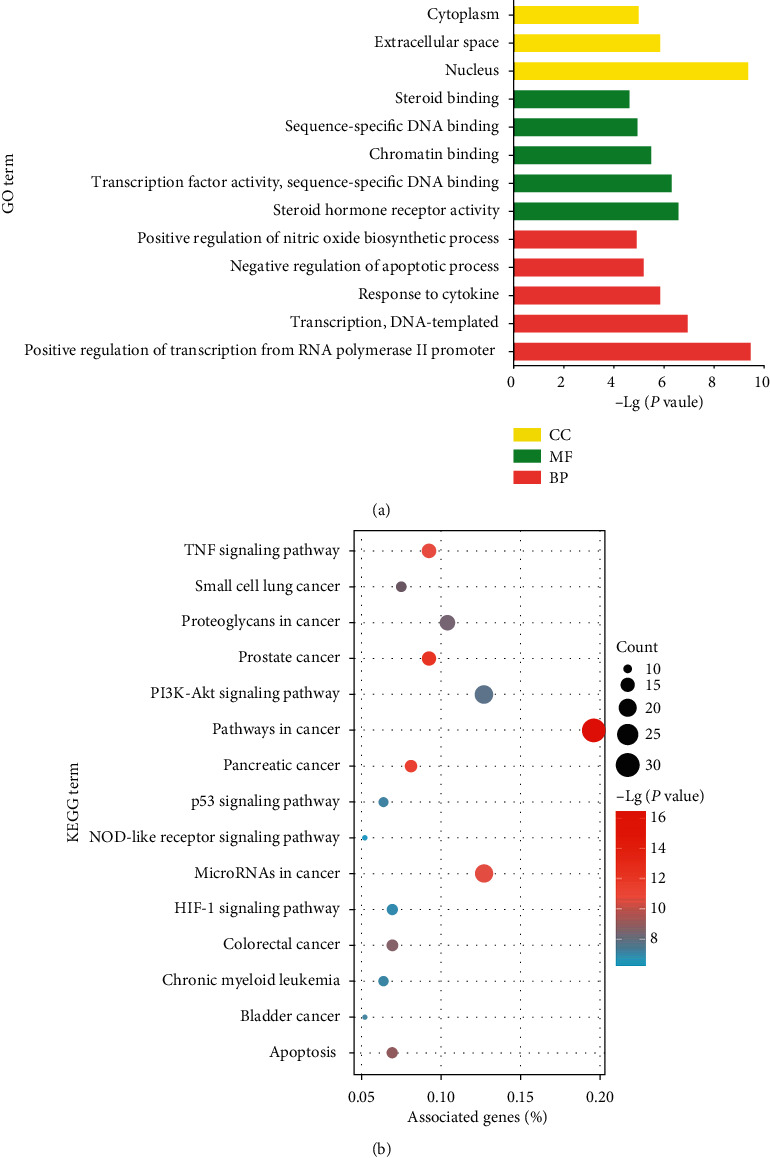
Functional enrichment analysis of therapeutic targets of the CHs for CRC. (a) The five most significantly enriched BP and MF terms. For CC, only three terms were significantly enriched. (b) The top 15 most significantly enriched KEGG terms. The bubble size represents the count of therapeutic targets enriched in a certain pathway; the abscissa shows the percentage of target genes to the background genes of a certain pathway.

Functional enrichment analysis was performed for 118 therapeutic targets. In total, 17 GO terms were identified, including 8 biological process (BP) terms, 6 molecule function (MF) terms, and 3 cellular component (CC) terms (*P* value and FDR both <0.05). For BP, the targets mainly participated in the regulation of transcription, apoptosis, and response to cytokine. For MF, the targets were mainly responsible for the activity of steroid hormone receptor and transcription factor, as well as binding of chromatin, DNA, and steroid. For CC, the targets were mainly distributed in nucleus, extracellular space, and cytoplasm. The five most significantly enriched GO terms are shown as bar plots in Figure 3(a).

Besides, 44 KEGG pathway terms were enriched, many of which were pathways of specific kinds of cancer. Moreover, microRNAs in cancer and tumor necrosis factor (TNF), apoptosis, PI3K-Akt, and p53 signaling pathways, and so on were enriched. The top fifteen most significantly enriched KEGG terms are shown as a bubble chart in Figure 3(b).

### 3.4. The Network of Herb-Bioactive Compound-Therapeutic Target

The network of herb-bioactive compound-therapeutic target was composed of 277 nodes (10 herbs, 149 bioactive compounds, and 118 targets) and 1240 edges (Figure 3). Some bioactive compounds were absent in the network, in default of interaction with any therapeutic target. Among 149 compounds, quercetin might act on the most targets (degree = 59), followed by wogonin (degree = 33) and luteolin (degree = 25), the cumulative number of targets of the above three compounds was 82 (over 55% of all therapeutic targets). The information of the top 15 bioactive compounds with the most interactive therapeutic targets is shown in Table 2.

### 3.5. PPI Network and Major Targets Screening

**Figure 4:**
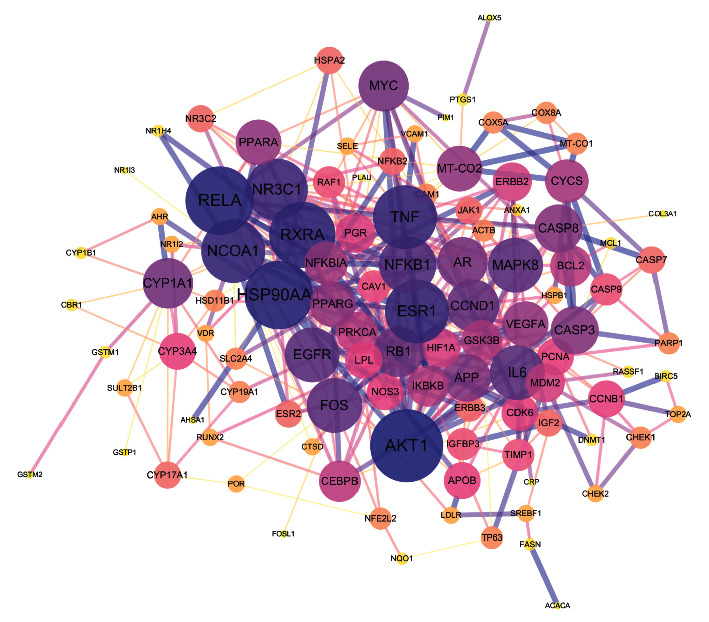
The PPI network of the therapeutic targets. The size and the color of nodes are proportional to their degree values; small size and bright color represent low degree values. The thickness and the color of edges are proportional to the interaction levels between nodes.

Based on the PPI analysis from the STRING, 12 targets were not involved in protein interactions; thus, the final PPI network consisted of 106 nodes (targets) and 364 edges (Figure 4). The median degree value of the PPI network was five, and 25 targets met the criteria of major targets in this network. The major targets interacted extensively with other proteins, suggesting their crucial roles in the development and progression of CRC.  Table 3 lists the detailed information on these major targets.

### 3.6. Prognostic Significance of Major Target Genes

**Figure 5:**
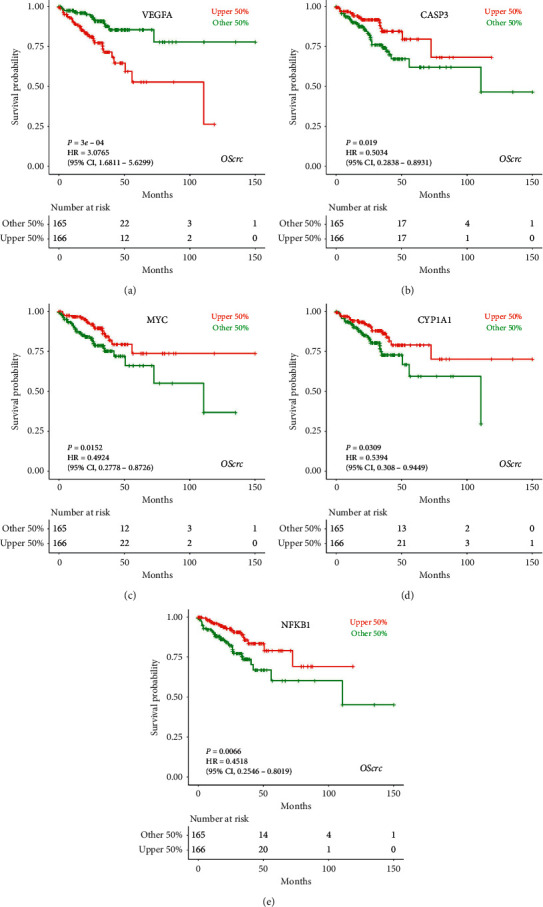
Five key target genes with prognostic significance for DFS in CRC patients. KM survival curves for (a) VEGFA, (b) CASP3, (c) MYC, (d) CYP1Y1, and (e) NFKB1.

Survival analysis was performed for every major target gene using the LOGpc server. The results showed that none of the major target genes showed significant association with OS in CRC patients. Nevertheless, high expression of VEGFA was associated with shorter DFS; high expression of vascular endothelial growth factor A (VEGFA), caspase 3 (CASP3), Myc protooncogene protein (MYC), cytochrome P450 enzymes 1A1 (CYP1A1), and NF-*κ*B p105/p50 subunit (NFKB1) were associated with longer DFS in CRC patients. So, these five targets were chosen as the key targets of the CHs treating CRC; the KM survival curves of them are presented in Figure 5. And they were selected to perform molecular docking simulation.

### 3.7. Results of Molecular Docking

**Figure 6:**
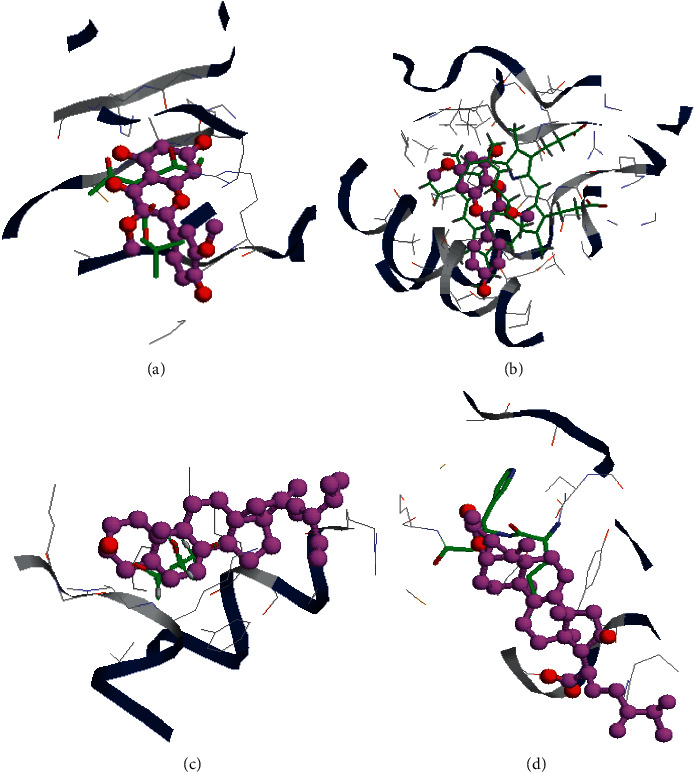
Molecular docking models of target proteins with bioactive compounds. (a) CASP3-MOL004961, (b) CYP1A1-MOL000239, (c) MYC-MOL004355, and (d) VEGFA-MOL000289. The original ligands are labeled on green color, and the predicted poses of bioactive compounds are labeled on pink color.

Because the NFKB1 protein-original ligand complex was unavailable from the PDB database, four key target proteins, VEGFA, CASP3, MYC, and CYP1Y1, were docked with 15 bioactive compound ligands. Because a lower fitness value signifies a stabler binding, the results of molecular docking implied four key targets might bind firmer with the 15 bioactive compounds, compared with their corresponding original ligands (Table 4). Furthermore, it is easy to find out that CYP1A1 protein has the best binding affinities with the 15 compound ligands.  Figure 6 shows the docking model of each target protein and the bioactive compound with the firmest binding.

## 4. Discussion

The current study could be divided into two major parts, which realized the inference from clinical experience to molecular mechanisms. In the first part, we identified the effective CHs in the treatment of CRC from abundant CHM prescriptions through data mining. In the second part, we deduced the therapeutic targets and mechanisms of the CHs acting on CRC by network pharmacology approach.

Firstly, ten Chinese herbs were identified as the CHs in the treatment of CRC, according to their high frequency of use and association rules with each other (Table 1 and Figure 2(a)). In CHM theory, Atractylodis Macrocephalae Rhizoma, Radix Astragali, Glycyrrhizae Radix et Rhizoma, Codonopsis Radix, and Angelicae Sinensis Radix are classified as tonic herbs to replenish the body's Qi (akin to the body's healthy energy) and blood. Poria, Coicis Semen, and Citrus Reticulatae Pericarpium are classified as dampness-dispelling herbs. Hedyotis Diffusae Herba and Scutellariae Barbatae Herba are classified as heat-clearing and detoxifying herbs. In the TCM theory of pathophysiology, deficiency of Qi and excess of toxic heat are the two most internal causes of CRC. What is more, deficiency of Qi usually leads to extra dampness, which is similar to the accumulation of metabolites in the body. Thus, the effects of the CHs were accorded with the TCM pathogenesis of CRC. Congruously, previous studies had demonstrated the benefits of the most CHs on CRC patients, including inhibiting side effects of chemotherapy and improving tumor response [38–41].

Then, the bioactive compounds and putative targets of the CHs were collected. The overlapping targets between the CHs and CRC were considered as the potential therapeutic targets of CHs for CRC (Figure 2(b)). The functional enrichment analysis uncovered the therapeutic targets might mainly participate in the regulation of transcription, binding of DNA, reacting to inflammation, and regulation of apoptosis (Figure 3(a)). And the involved signaling pathways were mostly cancer-related, including microRNAs, TNF, apoptosis, PI3K-Akt, and p53 pathways (Figure 3(b)).

MicroRNAs (miRNAs) participate in tumorigenesis, progression, invasion, and drug resistance in cancers, including CRC. The activation of the PI3K/Akt pathway in cancer promotion had been widely learned, and miRNAs can affect CRC by regulating PI3K/Akt pathway in dual ways. For example, miRNA21 and miRNA200c could promote CRC by downregulating phosphatase and tensin homolog (PTEN), a negative regulator of PI3K, while miRNA106a and miRNA1 could suppress CRC by activating PTEN or directly blocking PI3K/Akt pathway [42, 43]. Chronic inflammation is one of the characteristics of CRC. In the microenvironment of CRC, dense infiltrate of immune cells stimulates the secretion of proinflammatory cytokines, such as interleukin- (IL-) 6, IL-8, IL-1*β*, and TNF-*α*, which can synergistically activate signal transducer and activator of transcription 3 (STAT3), nuclear factor kappa-B (NF-*κ*B), and hypoxia-inducible factor 1*α* (HIF-1*α*) pathways, resulting in progression of CRC [44, 45]. As a well-known transcription factor and tumor suppresser, p53 protein drive cell apoptosis to avoid possibly cancer-inducing damaged DNA passing on to daughter cells. Not surprisingly, inactivation of the p53 pathway is often observed in CRC; binding of DNA to mutant p53 can amplify downstream protumor pathways [46].

**Figure 7:**
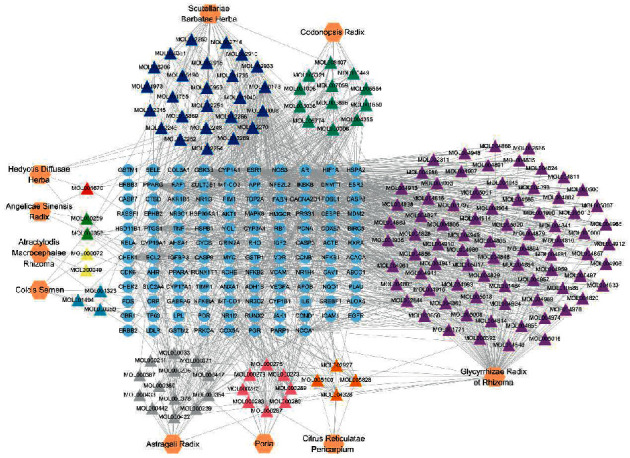
The herb-bioactive compound-therapeutic target network of the CHs. The hexagonal nodes represent the CHs, the triangle nodes represent the bioactive compounds, the circular nodes represent the therapeutic targets, and the edges represent the interactions among them.

Following, 15 compounds were recognized as the major bioactive compounds of the CHs in the treatment of CRC, with their importance in the herb-bioactive compound-therapeutic target network (Figure 7 and Table 2). Most major bioactive compounds belong to the flavonoid family, including quercetin, wogonin, luteolin, kaempferol, 5,7,4′-trihydroxy-8-methoxyflavone, formononetin, carthamidin, pinocembrin, and baicalein, all of which could be similarly used as chemopreventive agents, for their capacity of inducing cytotoxic apoptosis, anti-inflammation, antioxidation, and antiangiogenesis [47].

Quercetin was reported to induce cell cycle arrest and apoptosis, modulate estrogen receptors, alleviate oxidation, and inhibit angiogenesis in CRC [48]. Luteolin might suppress the proliferation and transformation of human CRC cells through antioxidation and miRNAs related pathways [49, 50]. Kaempferol could stimulate apoptosis in CRC cells, in which p53 mediated caspases activation might play critical roles [51]. And kaempferol should have a synergistic effect with 5-fluorouracil (5-FU) in CRC cell lines through PI3K/Akt inactivation [52]. Wogonin was observed to stimulate apoptosis through facilitating cytoplasmic localization of p53 in vitro, and its effects of reducing tumor multiplicity and preserving colon length were verified in vivo [53]. Another study demonstrated wogonin might simultaneously induce autophagy, apoptosis, and cell cycle arrest via blocking PI3K/Akt and STAT3 pathways [54], which was similar to formononetin [55]. Moreover, pinocembrin could trigger Bax-dependent mitochondrial apoptosis in CRC cells [56, 57]. Pachymic acid was also reported to induce cell cycle arrest and apoptosis in many kinds of digestive cancers [58–61].

Eburicoic acid exerts satisfying anti-inflammatory activity of relieving inflammatory cytokine production, and suppression of PI3K/Akt/mTOR/NF-*κ*B pathway might be involved [62, 63]. 5,7,4′-Trihydroxy-8-methoxyflavone might suppress inflammatory cytokine production both *in vitro* and *in vivo* [64]. Carthamidin might induce DNA damage and oxidative cell death in CRC cells [65]. Baicalein could repress CRC cells proliferation through lessening the ezrin gene's expression and increasing p53 pathway-related proteins [66], and it could suppress invasion of CRC cells by blocking extracellular regulated protein kinases (ERK) pathway [67].

Next, VEGFA, CASP3, MYC, CYP1Y1, and NFKB1 were identified as key targets of the CHs in the treatment of CRC, with their contributions in the PPI network (Figure 4) and prognostic significance in CRC patients (Figure 5). Angiogenesis is responsible for tumor vascularization degree, growth, and metastasis. VEGFA is acknowledged as a significant proangiogenic factor, which is frequently overexpressed in metastatic CRC patients [68]. Besides, miRNAs, such as miR-203, miR-497, and miR-26a, might resist CRC by targeting VEGFA [69, 70]. CASP3 activation triggers anticancer apoptosis and could be used as a marker of apoptosis-targeted treatment response [71]. MYC is a family of transcriptional regulators, containing c-Myc, n-Myc, and l-Myc in mammals. Deregulation of c-Myc exists in 70% of CRC, which could partly explain genome instability, tumorigenesis, and other malignant behaviors of CRC cells [72, 73]. CYP1A1 is used to be infamous for producing severe carcinogens. Although still controversial, CYP1A1 had been proposed more importance in detoxification, which might provide protective effects against some oral carcinogens in CRC patients [74]. NFKB1 belongs to NF-*κ*B family, which consists of a class of transcription factors working as central regulators of inflammatory pathways, cell proliferation, and apoptosis [75]. Activation of NF-*κ*B had been frequently observed in CRC patients, associated with worse outcomes [76]. Based on the above discussions, we could summarize VEGFA as an unfavorable factor, while CASP3, MYC, and CYP1Y1 as favorable factors in CRC, which was consistent with the results of survival analyses. In the contrary, our survival analysis showed that NFKB1 was correlated with longer DFS in CRC patients; for this, further researches are still needed.

In the end, the molecular docking simulation (Table 3 and Figure 6) showed a great majority of the 15 major bioactive compounds might bind firmly to VEGFA, CASP3, MYC, and especially CYP1Y1 proteins and thus afforded the possibility for them to exert their pharmacological activities. The molecular docking simulation strengthened all the above investigations to some extent, but experimental validations are still needed.

## 5. Conclusion

In summary, this study combined data mining and network pharmacology approach to identify ten the CHs, fifteen major bioactive compounds (quercetin, wogonin, luteolin, etc.), and five key therapeutic targets (VEGF, CASP3, MYC, CYP1Y1, and NFKB1) in the treatment of CRC. Additionally, the underlying mechanisms of action of the CHs might be apoptosis induction, transcription modulation, and inflammation suppression, and microRNAs, TNF, apoptosis, PI3K-Akt, and p53 signal pathways are participated. This study threw light on the anti-CRC mechanisms of the CHs in a holistic manner, which might provide novel insights for therapeutic strategies and further studies. However, further experiments are still necessary.

## Figures and Tables

**Table 1:** Usage frequency and counts of bioactive compounds of the CHs.

Core herbs	Frequency	Proportion of all prescriptions	Counts of bioactive compounds
Atractylodis macrocephalae rhizoma	98	0.87	4
Poria	80	0.71	7
Astragali radix	75	0.66	17
Glycyrrhizae radix et rhizoma	70	0.62	91
Codonopsis radix	63	0.56	18
Coicis semen	50	0.44	6
Citrus reticulatae pericarpium	46	0.41	6
Hedyotis diffusae herba	44	0.39	5
Angelicae sinensis radix	39	0.35	3
Scutellariae barbatae herba	34	0.30	30

**Table 2:** Information of the top 15 bioactive compounds of the CHs.

Mol ID	Compound name	OB	DL	Count of therapeutic targets	Source of the CHs
MOL000098	Quercetin	46.43	0.28	59	Astragali radix, Hedyotis diffusae herba, scutellariae barbatae herba, glycyrrhizae radix et rhizoma
MOL000173	Wogonin	30.68	0.23	33	Scutellariae barbatae herba
MOL000006	Luteolin	36.16	0.25	25	Scutellariae barbatae Herba, Codonopsis radix
MOL000287	Eburicoic acid	38.7	0.81	25	Poria
MOL000422	Kaempferol	41.88	0.24	25	Astragali Radix, glycyrrhizae radix et rhizoma
MOL002933	5,7,4′-Trihydroxy-8-methoxyflavone	36.56	0.27	24	Scutellariae barbatae herba
MOL004961	Quercetin der.	46.45	0.33	23	Glycyrrhizae radix et rhizoma
MOL010586	Formononetin	66.39	0.21	23	Glycyrrhizae radix et rhizoma
MOL000239	Jaranol	50.83	0.29	20	Astragali Radix, glycyrrhizae radix et rhizoma
MOL000280	Dehydrotumulosic acid	31.07	0.82	20	Poria
MOL000289	Pachymic acid	33.63	0.81	19	Poria
MOL002910	Carthamidin	41.15	0.24	19	Scutellariae barbatae herba
MOL002844	Pinocembrin	64.72	0.18	18	Glycyrrhizae radix et rhizoma
MOL002714	Baicalein	33.52	0.21	16	Scutellariae barbatae herba
MOL004355	Spinasterol	42.98	0.76	16	Codonopsis radix

**Table 3:** The major targets of the CHs in the treatment of CRC.

No.	Target name	Gene symbol	Degree value in the PPI network
1	RAC-alpha serine/threonine-protein kinase	Akt1	23
2	Heat shock protein HSP 90-alpha	HSP90AA1	21
3	Transcription factor p65	RELA	21
4	Retinoic acid receptor RXR-alpha	RXRA	20
5	Tumor necrosis factor	TNF	19
6	Nuclear receptor coactivator 1	NCOA1	19
7	Estrogen receptor	ESR1	19
8	Glucocorticoid receptor	NR3C1	18
9	Nuclear factor NF-kappa-B p105 subunit	NFKB1	16
10	Epidermal growth factor receptor	EGFR	15
11	Mitogen-activated protein kinase 8	MAPK8	15
12	Proto-oncogene c-Fos	FOS	15
13	Interleukin-6	IL6	15
14	G1/S-specific cyclin-D1	CCND1	14
15	Cytochrome P450 1A1	CYP1A1	13
16	Retinoblastoma-associated protein	RB1	13
17	Myc protooncogene protein	MYC	13
18	Androgen receptor	AR	13
19	Caspase-8	CASP8	12
20	Caspase-3	CASP3	12
21	Amyloid beta A4 protein	APP	12
22	Cytochrome c oxidase subunit 2	MT-CO2	11
23	Peroxisome proliferator-activated receptor gamma	PPARG	11
24	Vascular endothelial growth factor A	VEGFA	11
25	Peroxisome proliferator-activated receptor alpha	PPARA	11

**Table 4:** The results of molecular docking simulation.

Target protein (PDB ID)	Ligand	Fitness (kcal/mol)	Target protein (PDB ID)	Ligand	Fitness (kcal/mol)
CASP3 (3GJR)	MOL004961	−120.03	MYC (5i4z)	MOL004355	−88.88
	MOL000098	−110.49		MOL000287	−88.70
	MOL000422	−110.21		MOL004961	−88.20
	MOL000006	−109.54		MOL000239	−85.61
	MOL002714	−107.83		MOL000098	−82.74
	MOL000289	−104.90		MOL000289	−82.22
	MOL000280	−101.64		MOL002933	−80.59
	MOL000239	−100.51		MOL000173	−80.46
	MOL000287	−99.34		MOL000280	−79.61
	MOL000173	−97.94		MOL000006	−79.57
	MOL002844	−97.52		MOL002714	−79.00
	MOL002933	−96.42		MOL000422	−77.54
	MOL002910	−96.29		MOL002910	−77.38
	MOL010586	−88.88		MOL010586	−76.89
	MOL004355	−87.31		MOL002844	−73.26
	DZE^*∗*^	−66.14		GOL^*∗*^	−44.23

CYP1A1 (6dwm)	MOL000239	−158.75	VEGFA (6d3o)	MOL000289	−102.14
	MOL000280	−156.15		MOL000280	−92.96
	MOL004961	−153.75		MOL000098	−91.14
	MOL002933	−151.42		MOL002910	−91.00
	MOL000006	−150.57		MOL000006	−90.53
	MOL000098	−147.94		MOL004961	−90.53
	MOL000422	−146.91		MOL000239	−82.89
	MOL002714	−144.12		MOL004355	−82.03
	MOL000287	−141.47		MOL002844	−81.16
	MOL004355	−140.61		MOL002933	−80.73
	MOL000173	−137.33		MOL000287	−80.18
	MOL000289	−135.84		MOL000422	−80.08
	MOL010586	−133.70		MOL002714	−77.53
	MOL002910	−119.95		MOL000173	−76.44
	MOL002844	−114.64		MOL010586	−70.71
	CPS^*∗*^	−107.82		NLE^*∗*^	−69.12

^*∗*^DZE, CPS, GOL, and NLE are the names of the original ligands in the PDB database.

## Data Availability

The information of the clinical studies included in this study is provided in Supplementary Table 1. All data that support the findings of this study are publicly available from the databases mentioned in the Materials and Methods section.

## Supplementary Materials

Supplementary Materials A list of the clinical studies included in this study is provided in Supplementary Table 1.
