# Supplementary material for: Mechanisms of Core Chinese Herbs against Colorectal Cancer: A Study Based on Data Mining and Network Pharmacology
Source: Evid Based Complement Alternat Med. 2020 Oct 27;2020:8325076. doi: 10.1155/2020/8325076 (PMC7641702; doi:10.1155/2020/8325076)
Supplement: Supplementary Materials — A list of the clinical studies included in this study is provided in Supplementary Table 1. [file 8325076.f1.docx]

**Supplementary Table 1**: The information of the clinical studies included in the current study.

| Database | Number of CHM prescription | Source of the clinical study (title of the article) | Journal |
| --- | --- | --- | --- |
| CNKI  (published in Chinese) | 1 | Clinical application of Jianpi Yiqi Decoction in patients with colorectal cancer during perioperative period | Academic Journal of Shanghai University of Traditional Chinese Medicine |
|  | 2 | Effect of traditional Chinese medicine on immune function and bone marrow suppression in patients with colorectal cancer undergoing chemotherapy | Chinese Journal of Public Health Engineering |
|  | 3 | Effect of Xiangsha Liujunzi Decoction on quality of life of patients with colorectal cancer after chemotherapy | Clinical Journal of Chinese Medicine |
|  | 4 | Clinical observation of Bazhen Decoction in the treatment of postoperative patients with colorectal cancer | Clinical Journal of Chinese Medicine |
|  | 5 | Effect of modified Siling powder combined with XELOX chemotherapy on cellular immune function and quality of life in patients with spleen deficiency and damp heat syndrome after colorectal cancer surgery | Modern Journal of Integrated Traditional Chinese and Western Medicine |
|  | 6 | Effect of Fuzheng Quxie anticancer therapy on postoperative survival and immune indexes of patients with colorectal cancer | Journal of Preventive Medicine of Chinese People's Liberation Army |
|  | 7 | Effect of Jianpi Yiqi method combined with raltitrexed chemotherapy on clinical efficacy and immune function of patients with advanced colorectal cancer | Modern Digestion & Intervention |
|  | 8 | Clinical study of Jianpi Liqi decoction combined with FOLFOX6 regimen in the treatment of middle and advanced rectal cancer with spleen deficiency and qi stagnation syndrome | China Modern Doctor |
|  | 9 | Jiedu Qingchang Kangai recipe in the treatment of 53 cases of primary colorectal cancer after operation | Zhejiang Journal of Traditional Chinese Medicine |
|  | 10 | Clinical study on the treatment of rectal cancer with Tongxia Zhuyu method | Shaanxi Journal of Traditional Chinese Medicine |
|  | 11 | Effect of Jianpi Fuzheng Recipe on T cell subsets, tre cells and quality of life in patients with colorectal cancer undergoing postoperative chemotherapy | Guiding Journal of Traditional Chinese Medicine and Pharmacy |
|  | 12 | Effect of fuzhengpeiben combined with irinotecan chemotherapy on Serum CA19-9 and MMP-7 levels in patients with advanced colorectal cancer | Beijing Journal of Traditional Chinese Medicine |
|  | 13 | Clinical study of Jianpi Huoxue Yiqi Decoction Combined with XELOX regimen in the treatment of advanced rectal cancer | Journal of Sichuan of Traditional Chinese Medicine |
|  | 14 | Clinical study of Xiaoyao Powder in the treatment of advanced colorectal cancer with stagnation of liver Qi | Guiding Journal of Traditional Chinese Medicine and Pharmacy |
| CNKI  (published in Chinese) | 15 | Effect of Shenlingbaizhu powder on immune function of postoperative chemotherapy in patients with colorectal cancer | Clinical Journal of Traditional Chinese Medicine |
|  | 16 | Effect of Fuzheng Guben decoction combined with FOLFOX chemotherapy on CD4 ~ + CD45RA ~ + T level in postoperative peripheral blood of patients with colorectal cancer | Modern Journal of Integrated Traditional Chinese and Western Medicine |
|  | 17 | Efficacy of Jingqi Shengbai decoction combined with FOLFOX6 regimen in adjuvant chemotherapy for rectal cancer | Medical Science Journal of Central South China |
|  | 18 | Effect of Zhenqi Fuzheng decoction combined with FOLFOX6 regimen in the treatment of elderly patients with advanced rectal cancer and its influence on immune function | Journal of Sichuan of Traditional Chinese Medicine |
|  | 19 | Clinical study of Guishao Liujunzi Decoction in the treatment of 25 cases of colorectal cancer with liver metastasis | Jiangsu Journal of Traditional Chinese Medicine |
|  | 20 | Fuzheng Kangai ointment combined with chemotherapy in the treatment of 54 cases of colorectal cancer | Fujian Journal of Traditional Chinese Medicine |
|  | 21 | Clinical effect of Yiqi Fusheng Decoction on postoperative chemotherapy of colorectal cancer | Academic Journal of Shanghai University of Traditional Chinese Medicine |
|  | 22 | Observation on the curative effect of zengxiaodu Decoction on postoperative chemotherapy of rectal cancer of qi stagnation and blood stasis type | Clinical Journal of Chinese Medicine |
|  | 23 | Quality of life of patients with colorectal cancer treated by Tongfu Jiedu decoction combined with chemotherapy and its role in regulating immune mechanism | Chinese Archives of Traditional Chinese Medicine |
|  | 24 | Treatment of 31 cases of advanced colorectal cancer with guishaoliujun decoction combined with raltitrexed chemotherapy | Zhejiang Journal of Traditional Chinese Medicine |
|  | 25 | Effect of modified Sijunzi Decoction on quality of life of patients with spleen stomach qi deficiency syndrome after colorectal cancer surgery | Journal of Community Medicine |
|  | 26 | Effect of Kangai No.2 prescription on immune function and quality of life of colorectal cancer patients with deficiency of Qi and blood | Occupation and Health |
|  | 27 | Analysis of Qinghua Decoction in treatment of postoperative damp heat syndrome of colorectal cancer and its influence on quality of life | Liaoning Journal of Traditional Chinese Medicine |
|  | 28 | Treatment of 62 cases of colorectal cancer with Jianpi Jiedu decoction combined with chemotherapy | Traditional Chinese Medicinal Research |
|  | 29 | Shenlingbaizhu powder combined with chemotherapy in the treatment of advanced colorectal cancer: a randomized parallel controlled study | Occupation and Health |
|  | 30 | Clinical study on Application of Bushen Jianpi method in perioperative period of colorectal cancer | Guangming Journal of Chinese Medicine |
|  | 31 | Clinical effect analysis of chemotherapy combined with traditional Chinese medicine in the treatment of colorectal cancer after operation | Clinical Journal of Chinese Medicine |
|  | 32 | Effect of Jianpi Qingchang Recipe on recurrence of colorectal adenoma after resection | Journal of Nanjing University of Traditional Chinese Medicine |
| CNKI  (published in Chinese) | 33 | Evaluation of the effect of Invigorating Spleen Method on improving the quality of life of patients with colorectal cancer with spleen deficiency syndrome after postoperative chemotherapy | Modern Journal of Integrated Traditional Chinese and Western Medicine |
|  | 34 | Clinical effect of chemotherapy combined with traditional Chinese medicine on postoperative colorectal cancer | China Modern Doctor |
|  | 35 | Clinical evaluation of Jianpi Yiqi Jiedu decoction combined with chemotherapy in the treatment of advanced colorectal cancer | Progress in Modern Biomedicine |
|  | 36 | Clinical observation of Yiqi Jiedu Xiaoai decoction combined with chemotherapy in the treatment of advanced metastatic colorectal cancer with spleen deficiency and Blood Stasis Toxin syndrome | Chinese Journal of Experimental Traditional Medical Formulae |
|  | 37 | Clinical observation of traditional Chinese medicine combined with XELOX regimen in the first line treatment of elderly patients with advanced colorectal cancer | Modern Journal of Integrated Traditional Chinese and Western Medicine |
|  | 38 | Clinical study on Banxia Xiexin Decoction in the treatment of liver spleen disharmony after colorectal cancer operation | World Chinese Medicine |
|  | 39 | Effect of Qifu longkui Decoction on serum vascular endothelial growth factor and immune function in patients with advanced rectal cancer | Jilin Journal of Chinese Medicine |
|  | 40 | Effect of Fuzheng Xiaoai decoction combined with chemotherapy on immune function, clinical symptoms and quality of life in patients with colorectal cancer | Chinese Archives of Traditional Chinese Medicine |
|  | 41 | Clinical study on 30 cases of advanced refractory colorectal cancer treated with "changfufang" | Jiangsu Journal of Traditional Chinese Medicine |
|  | 42 | Clinical observation of Jianpi Huoxue traditional Chinese medicine combined with chemotherapy in the treatment of postoperative patients with colorectal cancer | Journal of New Chinese Medicine |
|  | 43 | Effect of Yiqi Jiedu Decoction on tumor markers and immune function in patients with colorectal cancer | Journal of Sichuan of Traditional Chinese Medicine |
|  | 44 | Effect and effect of Jianpi Yiai Decoction on recurrence and metastasis of colorectal cancer after operation | Journal of Sichuan of Traditional Chinese Medicine |
|  | 45 | Effect of Jianpi Yiqi Huoxue Method Combined with chemotherapy on the curative effect and quality of life of postoperative patients with colorectal cancer | Modern Journal of Integrated Traditional Chinese and Western Medicine |
|  | 46 | Clinical observation of Shenlingbaizhu powder in adjuvant treatment of postoperative chemotherapy in patients with colorectal cancer | Chinese Traditional Patent Medicine |
|  | 47 | Yiqi Huoxue Decoction Combined with chemotherapy in the treatment of advanced colorectal cancer: a randomized parallel controlled study | Journal of Practical Traditional Chinese Internal Medicine |
|  | 48 | Effect of invigorating spleen and removing blood stasis on gastrointestinal function in patients with colorectal cancer after chemotherapy | Chinese Journal of Gerontology |
|  | 49 | Clinical observation of Shenlingbaizhu powder in adjuvant chemotherapy for advanced colorectal cancer | China Pharmacy |
| CNKI  (published in Chinese) | 50 | Clinical study of three-step cycle therapy combined with chemotherapy in the treatment of stage Ⅳ colorectal cancer* | Journal of Sichuan of Traditional Chinese Medicine |
|  | 51 |  |  |
|  | 52 |  |  |
|  | 53 | Effect of traditional Chinese medicine combined with chemotherapy on the expression of CEA, CD3 and CD8 in patients with colorectal cancer | The Practical Journal of Cancer |
|  | 54 | Clinical observation of Jianpi Jiedu decoction combined with chemotherapy in the treatment of postoperative patients with colorectal cancer | Journal of Hunan University of Chinese Medicine |
|  | 55 | Clinical study of FOLFOX4 regimen combined with Yiqi Jianpi Decoction in the treatment of colorectal cancer | The Chinese Journal of Clinical Pharmacology |
|  | 56 | Clinical effect of Shaoyao decoction combined with conventional western medicine in the treatment of advanced colorectal cancer | Chinese Journal of Clinical Rational Drug Use |
|  | 57 | Clinical effect of Yiqi Yangxue mixture on bone marrow suppression induced by chemotherapy for metastatic colorectal cancer | Hebei Medical Journal |
|  | 58 | Clinical study on Jianpi Anchang decoction combined with chemotherapy in the treatment of advanced colorectal cancer | Zhejiang Journal of Integrated Traditional Chinese and Western Medicine |
|  | 59 | Effect and mechanism analysis of Jianpi Yishen therapy on cancer-related fatigue in patients with advanced colorectal cancer | Chinese Journal of Experimental Traditional Medical Formulae |
|  | 60 | Clinical study on Jianpi Xiaoai decoction combined with chemotherapy in the treatment of advanced metastatic colorectal cancer | China Journal of Traditional Chinese Medicine and Pharmacy |
|  | 61 | Study on Zibu decoction combined with capecitabine in the treatment of advanced colorectal cancer with Qi and blood deficiency syndrome after first-line chemotherapy | Modern Journal of Integrated Traditional Chinese and Western Medicine |
|  | 62 | Effect of Jianpi Fuzheng Quxie Recipe on clinical efficacy and prognosis of postoperative patients with colon cancer | Liaoning Journal of Traditional Chinese Medicine |
|  | 63 | Clinical study on adjuvant treatment of colorectal cancer with traditional Chinese Medicine | Journal of Clinical Medicine in Practice |
|  | 64 | Clinical observation of Jianpi Anchang Decoction in the treatment of advanced colorectal cancer | Zhejiang Journal of Traditional Chinese Medicine |
|  | 65 | Effect of traditional Chinese medicine combined with FOLFOX4 regimen in the treatment of postoperative colorectal cancer | China Modern Doctor |
|  | 66 | Clinical observation of chemotherapy combined with traditional Chinese medicine decoction in the treatment of postoperative colorectal cancer with Qi blood deficiency | Electronic Journal of Clinical Medical Literature |
|  | 67 | Efficacy and safety of Tongtai mixture combined with xeliri regimen in the treatment of advanced colorectal cancer | Modern Digestion & Intervention |
| CNKI  (published in Chinese) | 68 | Clinical observation of chemotherapy combined with traditional Chinese medicine in the treatment of colorectal cancer | Journal of Medical Forum |
|  | 69 | Effect of Fuzheng No.1 Decoction on immune function of postoperative patients with colorectal cancer | Journal of Colorectal & Anal Surgery |
|  | 70 | Observation on the curative effect of compound Chinese medicine combined with chemotherapy on advanced colorectal cancer | Journal of Liaoning University of Traditional Chinese Medicine |
|  | 71 | Short term clinical effect of Fuzheng Jiedu Decoction on patients with colorectal cancer after radical operation | Liaoning Journal of Traditional Chinese Medicine |
|  | 72 | Clinical observation on treating 63 cases of senile colorectal cancer of damp heat accumulation type with Changyi recipe | Practical Geriatrics |
|  | 73 | Effect of Jianpi Qingchang method on cytokines in colorectal cancer patients with intestinal barrier dysfunction induced by chemotherapy | China Journal of Traditional Chinese Medicine and Pharmacy |
|  | 74 | Clinical observation of self-made traditional Chinese medicine combined with chemotherapy in the treatment of advanced colorectal cancer | Chinese Journal of Traditional Medical Science and Technology |
|  | 75 | Effect of Yiqi Yangyin traditional Chinese medicine combined with FOLFOX4 chemotherapy on immune function of postoperative patients with colorectal cancer | Modern Digestion & Intervention |
|  | 76 | Clinical observation of self-made compound Chinese medicine combined with tegafur in the treatment of advanced colorectal cancer | Asia-Pacific Traditional Medicine |
|  | 77 | Clinical observation on 21 cases of advanced colorectal cancer treated with integrated traditional Chinese and Western Medicine | Journal of New Chinese Medicine |
|  | 78 | Effect analysis of Yiqi Jianpi traditional Chinese medicine combined with chemotherapy on patients with advanced colorectal cancer | Clinical Journal of Chinese Medicine |
|  | 79 | Effect of Jianpi Bushen formula on postoperative adjuvant treatment of colorectal cancer | Shanghai Medical Journal |
|  | 80 | Effect analysis of combined application of Jianpi Jiedu traditional Chinese medicine prescription and double pathway chemotherapy in the treatment of colorectal cancer | Contemporary Medical Symposium |
|  | 81 | Clinical observation of Jiedu Qingchang Decoction in the treatment of 28 cases of advanced colorectal cancer | Heilongjiang Journal of Traditional Chinese Medicine |
|  | 82 | Effect of Lingnan traditional Chinese medicine Longzhu Xiaoliu Decoction on immune suppression microenvironment of colorectal cancer | Journal of New Chinese Medicine |
|  | 83 | Clinical observation on 28 cases of advanced colorectal cancer treated with traditional Chinese medicine combined with chemotherapy | Zhejiang Journal of Traditional Chinese Medicine |
|  | 84 | Clinical observation of Jianpi Xiaoai Decoction in the treatment of elderly patients with advanced colorectal cancer | Chinese Journal of Information on Traditional Chinese Medicine |
|  | 85 | Effect of Fuzheng anticancer therapy on survival time and quality of life in patients with advanced metastatic colorectal cancer | Academic Journal of Shanghai University of Traditional Chinese Medicine |
| CNKI  (published in Chinese) | 86 | Clinical study of Jianpi Huayu method in preventing recurrence and metastasis of colorectal cancer after chemotherapy | Liaoning Journal of Traditional Chinese Medicine |
|  | 87 | Clinical observation of Quyu Jiedu method combined with tegafur in the treatment of elderly patients with poor physical score or liver metastasis from colorectal cancer | Guide of China Medicine |
|  | 88 | Clinical study on Jianpi Jiedu decoction combined with capecitabine tablets in the treatment of advanced colorectal cancer | Academic Journal of Shanghai University of Traditional Chinese Medicine |
|  | 89 | Clinical observation of Zibu Decoction in the treatment of elderly patients with advanced colorectal cancer of Qi and blood deficiency type | World Chinese Medicine |
|  | 90 | Clinical study on the effect of modified Tenglong Buzhong Decoction on immune function of patients with colorectal cancer | World Journal of Integrated Traditional and Western Medicine |
|  | 91 | Clinical observation of Shengbai Decoction in the treatment of leukopenia after colorectal cancer surgery | Chinese Journal of Modern Drug Application |
|  | 92 | Clinical observation on 32 cases of postoperative colorectal cancer treated with traditional Chinese medicine and FOLFIRI chemotherapy | Guide of China Medicine |
|  | 93 | Clinical observation of compound Changtai combined with FOLFIRI chemotherapy in the treatment of advanced colorectal cancer | Contemporary Medicine |
|  | 94 | Effect of Fuzheng Jianpi Decoction Combined with chemotherapy on advanced colorectal cancer | Chinese Archives of Traditional Chinese Medicine |
|  | 95 | Clinical study of yichangning decoction combined with folfox-4 regimen in the treatment of 30 cases of advanced colorectal cancer | Fujian Journal of Traditional Chinese Medicine |
|  | 96 | Clinical observation of spleen invigorating traditional Chinese medicine combined with chemotherapy in the treatment of advanced colorectal cancer | Shanghai Journal of Traditional Chinese Medicine |
|  | 97 | 45 cases of advanced colorectal cancer treated with Yiqi Jiedu decoction combined with capeox regimen | Shanghai Journal of Traditional Chinese Medicine |
|  | 98 | Effect of Jianpi Jiedu Decoction on quality of life in patients with advanced colorectal cancer | Hebei Journal of Traditional Chinese Medicine |
|  | 99 | Clinical observation of Jianpi compound combined with chemotherapy in the treatment of postoperative patients with colorectal cancer | Journal of Clinical Medicine in Practice |
|  | 100 | Clinical study on the intervention of traditional Chinese medicine during the period of postoperative chemotherapy for colorectal cancer | Guangming Journal of Chinese Medicine |
|  | 101 | Clinical observation of Zhao's fine tune No.3 combined with chemotherapy in the treatment of colorectal cancer | Lishizhen Medicine and Materia Medica Research |
|  | 102 | Clinical observation of jiangniling combined with chemotherapy in the treatment of advanced colorectal cancer | China Medical Herald |
|  | 103 | Treatment of 47 cases of advanced colorectal cancer with Jianpi Yiqi traditional Chinese medicine combined with chemotherapy | Journal of Chinese Oncology |
|  | 104 | Clinical observation on the effect of invigorating spleen herbs on the survival period of colorectal cancer patients with liver metastasis | Liaoning Journal of Traditional Chinese Medicine |
|  | 105 | Clinical analysis of TCM treatment of colorectal cancer patients with postoperative chemotherapy | Chinese Journal of General Practice |
| CNKI  (published in Chinese) | 106 | Clinical observation of Bazhen decoction combined with chemotherapy in the treatment of 31 cases of advanced colorectal cancer | Hebei Journal of Traditional Chinese Medicine |
|  | 107 | Clinical observation on the treatment of elderly patients with advanced colorectal cancer by Integrated Traditional Chinese and Western Medicine | China & Foreign Medical Treatment |
|  | 108 | Clinical observation of changyijian in Treating 50 cases of postoperative colorectal cancer | Zhejiang Journal of Traditional Chinese Medicine |
|  | 109 | Clinical and experimental study of Fuzheng fangai oral liquid combined with chemotherapy in the treatment of advanced gastrointestinal cancer | Chinese Journal of Surgery of Integrated Traditional and Western Medicine |
|  | 110 | Clinical study on the treatment of advanced colorectal cancer pain with integrated traditional Chinese and Western Medicine | Journal of Zhejiang Chinese Medical University |
| MEDLINE  (published in English) | 111 | Chinese Medicine for Outcomes in Colorectal Cancer Patients: A Retrospective Clinical Study | Chin J Integr Med |
|  | 112 | Traditional Chinese Medicine Combined With Chemotherapy and Cetuximab or Bevacizumab for Metastatic Colorectal Cancer: A Randomized, Double-Blind, Placebo-Controlled Clinical Trial | Front Pharmacol |
|  | 113 | The Effect of Lon-Term Traditional Chinese Medicine Treatment on Survival Time of Colorectal Cancer Based on propensity Score Matchin: A Retrospective Cohort Study | Evid Based Complement Alternat Med |
